# Study on dynamic changes and correlations of microbial diversity, key enzyme activity and conventional components in tobacco leaves during the aging process

**DOI:** 10.1186/s40643-025-00969-3

**Published:** 2025-11-05

**Authors:** Wen Cai, Qian-ying Zhang, Peng-cheng Zhu, Wanrong Hu, Hongyue An, Dan-Qun Huo, Dongliang Li

**Affiliations:** 1 China Tobacco Technology Innovation Center for Cigar, China Tobacco Sichuan Industrial Co., Ltd, Chengdu, 610066 China; 2 College of Bioengineering, Chongqing University, Chongqing, 400000 China; 3 Technology Center, China Tobacco Sichuan Industrial Co., Ltd., Chengdu, 610066 China

**Keywords:** Aging process, Microbial diversity, Enzyme activity, Conventional components, Correlation analysis

## Abstract

This study focuses on the aging process of strong-flavor flue-cured tobacco leaves (SCTL) and light-flavor flue-cured tobacco leaves (FCTL), and explores the effects of microbial metabolism and enzyme action on tobacco leaf components and quality during this process. It employs methods including high-throughput sequencing, functional prediction of microbial communities via the PICRUSt2/MetaCyc database, dynamic analysis of conventional components and enzyme activities, and redundancy analysis. The results showed that under the same aging duration, the microbial community diversity structures of tobacco leaves with different quality grades were similar, and a total of 21 species of differential bacteria and 9 species of differential fungi were screened out; During the aging process, the contents of starch and total phenols in tobacco leaves decreased significantly, reducing sugars and total sugars showed a fluctuating trend, while total nitrogen and total plant alkaloids had no significant changes. Among them, the starch content in SCTL was higher than that in FCTL, while the contents of total phenols, reducing sugars and total sugars in SCTL were lower than those in FCTL; the contents of total nitrogen and total plant alkaloids were comparable between the two types of tobacco leaves. Additionally, the contents of reducing sugars, total sugars and starch in both types of tobacco leaves were positively correlated with tobacco leaf grade, while the contents of total phenols and total nitrogen were negatively correlated with tobacco leaf grade; The enzyme activities in the two types of tobacco leaves showed fluctuating changes during aging, and redundancy analysis (RDA) identified significant correlations between microbial taxa, enzyme activities and tobacco leaf compounds. This study provides a basis for microbial strain selection and precision fermentation strategies in tobacco aging.

**Graphical abstract:**

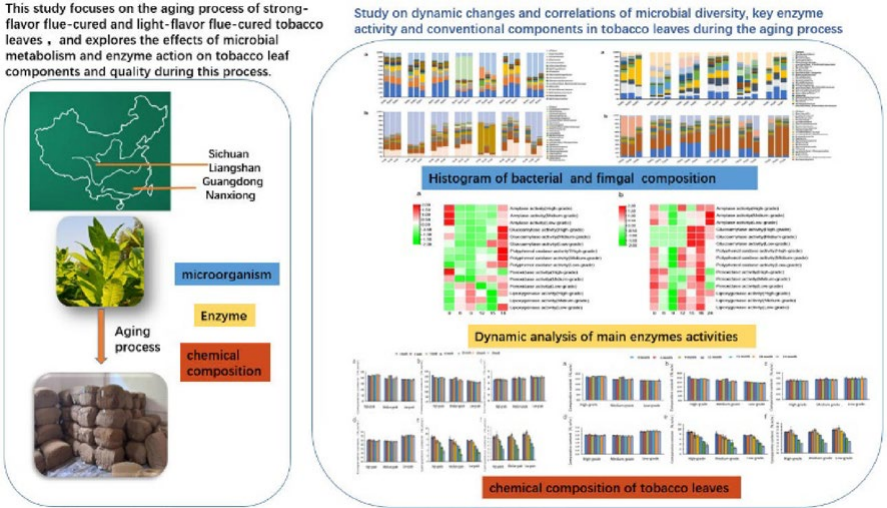

## Introduction

Tobacco is an important economic crop (Zhang et al. 2022). In the tobacco processing industry, aging is a critical step to improve the quality of tobacco leaves. Freshly harvested tobacco leaves typically exhibit characteristics such as strong green odor, high irritation, and weak aroma, necessitating aging before further use. Natural aging is the primary method, where flue-cured tobacco is stored in aging warehouses. Under natural conditions, through the combined action of microorganisms, enzymes, and chemical oxidation, macromolecular substances in the leaves are converted into small-molecule substances with specific flavors and aromas, thereby improving the quality of tobacco leaves (Zhang et al. 2023b, a, c). This study mainly takes tobacco leaves of two flavors and three grades as the research objects, which are strong-flavor flue-cured tobacco leaves (SCTL) and light-flavor flue-cured tobacco leaves (FCTL). Features of SCTL: The aroma is rich, strong and overflowing, with a caramel-sweet aroma charm, and the aroma sensing speed is relatively slow. The proportion of aroma components with high relative molecular weight in the smoke is relatively large. Features of FCTL: The aroma is full, elegant, natural and mellow, with the characteristic of sweet and fresh flue-cured tobacco aroma, accompanied by a certain amount of paste aroma, spicy aroma and woody aroma charm, giving people an elegant, fragrant, natural and fresh feeling.

Research on microorganisms during the aging process of tobacco leaves mainly focuses on two aspects. One is to explore the microbial groups present during the aging process and the roles they play (Liu et al. 2025, 2024; Shang 2025). The other is to isolate microorganisms from the surface of tobacco leaves, cultivate and screen them to improve the aging process of tobacco leaves and the aroma quality of tobacco leaves (Liang et al. 2023; Zhao et al. 2024). Enzymes are the core biological factors that promote the degradation of macromolecular substances and their conversion into flavor components during the aging process of tobacco leaves. The main sources of enzymes are, firstly, the plant enzymes with residual activity in tobacco after re—drying, and secondly, the enzymes secreted by the metabolism of tobacco leaf microorganisms. Enzymes with different functions act on different components of tobacco leaves, producing different quality—improving effects.Some studies have conducted dynamic monitoring of enzyme activities related to the degradation and transformation of components such as total sugar, total nitrogen, and starch in tobacco leaves (Chai et al. 2024; Zhang et al. 2014; Liu et al. 2015a, b; Wu et al. 2010). Tobacco quality is closely related to the composition and content of its chemical components (Zhang et al. 2014, Gong et al. 2010a, b, Xu et al. 2004). Only when the chemical components are balanced can tobacco have suitable aroma and taste. The content and ratio of main chemical components in tobacco determine the smoke characteristics of tobacco and its products, thus determining the industrial use value of tobacco (Liu et al. 2015a, b; Wu et al. 2013; Zhang et al. 2023b, a, c).

At present, previous studies have mainly focused on the research of the variation laws of tobacco leaf microorganisms and conventional chemical components during the aging process. The research on enzymology is more concentrated on treating tobacco leaves with biological enzymes, and there is little analysis of the correlation among the three. This study is the first to integrate microbial dynamics, enzymatic activity, and chemical composition in comparing strong-flavor and light-flavor flue-cured tobacco. We aimed to characterize microbial succession, measure enzymatic activity, and link these with chemical transformations during aging.

## Materials and methods

### Materials

SCTL is 2018 Mixed Threshed Tobacco Leaves (Grade C4F) from Nanxiong, Guangdong, FCTL is 2018 Mixed Threshed Tobacco Strips (Grade CXBK) from Liangshan, Sichuan. All tobacco leaf samples are natural aging. Details of the sampling schedule and environmental conditions are shown in Table 1. A five-point sampling method was employed, with samples collected from aging boxes of the same tobacco variety placed in three locations within the same warehouse. These three groups of samples constituted parallel samples. Sampling tools and packaging were ensured to be free of contamination. After collection, samples were stored in a − 20 °C refrigerator in the laboratory for later use.

**Table 1:** Base information of tobacco samples

Sample	Sampling time and conditions							
Natural aging time/month		0	6	9	12	15	18	24
SCTL	High-grade	Yes	Yes	Yes	Yes	Yes	Yes	Yes
	Mediu-grade	Yes	Yes	Yes	Yes	Yes	Yes	Yes
	Low-grade	Yes	Yes	Yes	Yes	Yes	Yes	Yes
Natural aging time/month		0	6	9	12	15	18	
FCTL	High-grade	Yes	Yes	Yes	Yes	Yes	Yes	
	Mediu-grade	Yes	Yes	Yes	Yes	Yes	Yes	
	Low-grade	Yes	Yes	Yes	Yes	Yes	Yes	
Conditions	Temperature,/℃	13	21.5	28	14	24	30	14
	Relative humidity /%	67	70	72	70	72	75	65

“Yes” indicates sample collection

### Chemical reagents and instruments

Q5™ High-Fidelity DNA polymerase, New England Biolabs, Inc. in the United States; DNA Marker, dNTP MIX, Beijing TransGen Biotech Co., Ltd.; DNA Gel Recovery Kit, Axygen Scientific, Inc., USA; QIAamp DNA Stool Mini Kit, Qiagen GmbH (Germany); Quant-iT PicoGreen dsDNA Assay Kit, Thermo Fisher Scientific (China) Co., Ltd.; Microbial Total DNA Extraction Kit, Omega Company in the United States; MiSeq Reagent Kit V3, Illumina, Inc., USA; DNA Library Prep Kit, UltraPure Agarose, Thermo Fisher Scientific (China) Co., Ltd.. The chemical reagents used in this experiment include chloro-L-phenylalanine, ribitol, tritiated naphthalene, C5-C30 n-alkanes, as well as organic solvents such as acetonitrile and methanol, salt substances like methoxylamine hydrochloride, potassium hydrogen phosphate, potassium dihydrogen phosphate, sodium chloride, and potassium iodide, and also organic reagents including pyridine, trifluoroacetamide, 3,5-dinitrosalicylic acid, phenol, anthrone, catechol, linoleic acid, guaiacol, soluble starch, and glucose; in addition, inorganic reagents such as hydrogen peroxide, sodium hydroxide, hydrochloric acid, and concentrated sulfuric acid, along with surfactants like Tween-80, are also required, All the aforementioned reagents are produced by Aladdin Reagent (Shanghai) Co., Ltd..

Eppendorf 5810 R Benchtop High-Speed Refrigerated Centrifuge, Eppendorf Company in Germany; Electrophoresis apparatus, American Bio-Rad Gel Imaging Analysis System, PowerPac Basic Power Supply 164–5050, Gel Doc XR + American Bole Company; Sequencing Platform Illumina MiSeqPE250, American Illumina company;Ultrapure Water System, Milli-Q Reference German Millipore company; Ultramicro Spectrophotometer NanoDrop One, Thermo Fisher Scientific (China) Co., Ltd.; Microplate reader FLx800, BioTek Corporation in the United States; Automatic High-pressure Sterilizer, Shanghai Boxun Medical and Biological Instrument Co., Ltd.; Constant Temperature Oscillating Incubator, ZHZY-C Shanghai Zhichu Instrument Co., Ltd.; Constant Temperature Water Bath, DK-BD type, Shanghai Jinghong Experimental Equipment Co., Ltd.; Fourier Transform Near-Infrared Spectrometer, MATRIX-F model, Bruker Optics GmbH, Germany; Grinding Instrument TL-48R, Shanghai Jingxin Industrial Development Co., Ltd.; Microplate reader FLx800, produced by BioTek (a company from the United States).

## Methods

### High-throughput sequencing

The high-throughput sequencing method based on Illumina MiSeq PE250 platform was used to study the microbial diversity of each tobacco sample during the aging process.

1. Collection of foliar microorganisms Refer to the method of Ye (Ye et al. 2017), weigh 10 ± 0.1 g of tobacco leaf samples, add 200 mL of sterile phosphate buffer solution (PBS) (0.1 mol/L, pH 7.2), oscillate at 30℃ and 20 r/min for 2 h, ultrasonicate for 5 min, and filter out the tobacco slices with sterile absorbent gauze. The filtrate is centrifuged at 10,000 × g for 10 min to collect the precipitate, which is the mixture of tobacco leaf microorganisms.
2. Extraction of total microbial DNA: Extract total microbial DNA according to the instructions of the extraction kit. Quantify the DNA with an ultramicro spectrophotometer, and detect the DNA extraction quality by gel electrophoresis (1.2% agarose). When the total DNA amount reaches more than 20 ng, it can meet the sequencing requirements.
3. Polymerase chain reaction (PCR) amplification of target fragments: The V4-V5 variable region of bacterial 16S rDNA and the ITS1-ITS4 variable region of fungi are amplified. The primer information is shown in Table 2.
4. Amplification system (25 μL): 5 μL of reaction buffer, 0.25 μL of Q5 DNA polymerase, 5 μL of GC buffer, 2 μL of dNTP (2.5 mmol/L), 1 μL of forward primer (10 μmol/L), 1 μL of reverse primer (10 μmol/L), 2 μL of DNA template, and 8.75 μL of ddH₂O.
5. Amplification conditions: Pre-denaturation at 98 °C for 2 min; denaturation at 98 °C for 30 s, annealing at 55 °C for 30 s, extension at 72 °C for 30 s, with 30 cycles of denaturation to extension; finally, extension at 72 °C for 5 min, and annealing at 10 °C for indefinite time.
6. Preparation of sequencing libraries and data processing: Entrusted to Shanghai Personalbio Technology Co., Ltd. The raw sequence data were denoised (including sequence quality control, denoising, splicing, and chimera removal) following the QIIME2 dada2 analysis pipeline (https://docs.qiime2.org/2019.7/tutorials/overview/) to obtain effective sequences.
7. Bioinformatics analysis: Effective sequences with 100% similarity were defined as one ASV (amplicon sequence variant). The bacterial 16S rRNA gene was subjected to BLAST alignment against the Silva database (Release 13.8, https://www.arb-silva.de/), and the fungal ITS1 gene against the UNITE database (Release 8.0, https://unite.ut.ee/) to annotate the biological classification information of ASVs. The sequences of each sample were randomly rarefied for ASVs using the rarefaction method, with the sequence depth level set as 95% of the minimum sample sequence count. The PICRUSt2 (Phylogenetic Investigation of Communities by Reconstruction of Unobserved States) software package (https://github.com/picrust/PICRUSt2/wiki) was used.

**Table 2:** Information of primer

Primer name	Forward primer sequence	Reverse primer sequence
Bacteria V4-V5	GTGCCAGCMGCCGCGGTAA	CCGTCAATTCMTTTRAGTTT
Fungus ITS1-ITS4	GGAAGTAAAAGTCGTAACAAGG	GCTGCGTTCTTCATCGATGC

#### Enzyme activity assays

Preparation of Crude Enzyme Extract: Take (2.5 ± 0.1) g of tobacco powder, add it to 30 mL of PBS, perform shaking extraction at room temperature for 1 h, centrifuge at 7000 r/min for 15 min, separate the supernatant, and obtain the crude enzyme extract.The enzyme activity of each sample is calculated based on the enzyme activity of the crude enzyme extract per gram of absolute dry tobacco powder (DW) (U/g).

Amylase can randomly break the α-1, 4 glycosidic bonds in starch molecules, causing the blue-violet color reaction of starch molecules with iodine to gradually disappear. The speed and degree of color disappearance are related to the enzyme activity.Detect the absorbance change of the system at a wavelength of 660 nm to calculate the enzyme activity.Definition of amylase activity: Under the conditions of 60 °C and pH 5.6, the amount of soluble starch hydrolyzed by 1 min of crude enzyme solution is defined as one enzyme activity unit (U).

Glucoamylase cleaves α-1, 4 glycosidic bonds from the non-reducing ends of starch molecular chains. The generated reducing sugars react with 3, 5-dinitrosalicylic acid (DNS) reagent to produce color. The absorbance change of the system is detected at a wavelength of 540 nm to calculate the enzyme activity.Definition of glucoamylase activity: Under the conditions of 40 °C and pH 4.6, the amount of glucose produced by 1 h of crude enzyme solution is defined as one enzyme activity unit (U), where 1 unit (U) is the amount of glucose (1 mg) generated by the crude enzyme solution within 1 h.

Lipoxygenase is a non-heme iron-containing oxygenase that specifically catalyzes linoleic acid with a cis,cis-1,4-pentadiene structure, leading to changes in absorbance. The spectrophotometric continuous determination method is used to dynamically detect the absorbance changes of the reaction system at a wavelength of 234 nm.Definition of lipoxygenase activity: Under the conditions of 30 °C and pH 6.0, an absorbance change of 0.1 per minute in the reaction system at 234 nm is defined as one enzyme activity unit (U).

Polyphenol oxidase is a copper-containing oxidase that, in the presence of oxygen, catalyzes the oxidation of catechol to form colored substances. The spectrophotometric continuous determination method is adopted to dynamically detect the absorbance changes of the reaction system at a wavelength of 410 nm. Definition of polyphenol oxidase activity: Under the conditions of 30 °C and pH 6.4, an absorbance change of 0.01 per minute in the reaction system at 410 nm is defined as one enzyme activity unit (U).

In the presence of hydrogen peroxide, peroxidase can oxidize guaiacol to generate a tea-brown substance. The spectrophotometric continuous determination method is used to dynamically detect the absorbance changes of the reaction system at a wavelength of 470 nm.Definition of peroxidase activity: Under the conditions of 30 °C and pH 6.9, an absorbance change of 0.01 per minute in the reaction system at 470 nm is defined as one enzyme activity unit (U).

### Routine chemical composition detection

Determination of Total Sugar and Reducing Sugar refer to 《YC/T 159–2019 Tobacco and Tobacco Products—Determination of Water-soluble Sugars—Continuous Flow Method* for the determination of total sugar and reducing sugar》.The determination of total nitrogen follows 《YCT 160–2018 Tobacco and Tobacco Products—Determination of Total Plant Alkaloids—Continuous Flow Method》.The determination of total plant alkaloids is conducted in accordance with 《YCT 161–2018 Tobacco and Tobacco Products—Determination of Total Nitrogen—Continuous Flow Method》.The starch content is determined by the anthrone colorimetry method with dilute acid hydrolysis.The total phenol content is determined by the Folin-Ciocalteu method.

### Data processing

All samples were determined using at least three parallel samples. The Canoco5.02 software was used for redundancy analysis of the correlation between dominant bacterial genera and temperature as well as relative humidity. R software (v3.6.2) was used to calculate Spearman's correlation coefficient and p-value, and Cytoscape (v3.7.1) software was used to draw the interaction network diagram of ASVs. Data are presented as mean ± standard deviation. GraphPad Prism software (v 8.0, San Diego, CA, USA) and Excel software (v 2017, Microsoft Office, US) were used for statistical analysis and graphic processing. Paired T-test was used for analysis of variance, and p < 0.05 was considered statistically significant. The unrestricted permutations model was adopted for permutation testing, and the P-values were corrected by the False Discovery Rate (FDR) to obtain the effect values of the correlation between dominant bacterial genera, main enzyme activities, and conventional chemical components, P < 0.05 indicates a significant correlation. The ecological data multivariate statistical analysis software Canoco (v5.02) was used to establish the correlation analysis between key factors.Differential analysis of sample data was performed using DPS data analysis software. Correlation analysis between dominant genera and enzyme activities was conducted using Canoco 5.02 software. Graphs were plotted using Origin 9.0, and visualization heatmaps were generated using R language.

## Results and analysis

### Analysis of microbial composition and functional prediction in tobacco leaves

#### Microbial diversity composition of tobacco leaves during aging process

In 63 samples (21 groups, 3 parallel samples per group) of SCTL, a total of 3,703,242 raw bacterial sequences were obtained, and 2,662,788 valid sequences were obtained after denoising; 3,925,130 raw fungal sequences and 3,441,733 valid sequences were obtained. ASVs (Amplicon Sequence Variants) were classified from valid sequences with 100% similarity. The mainstream analysis platform QIIME2 suggests that ASVs are more precise than the previously commonly used OTUs (operational taxonomic units). By annotating the species information of ASV representative sequences, the bacterial composition of three-grade SCTL at different aging stages was obtained, including microorganisms from 5 phyla, 26 classes, 28 orders, 273 families, and 887 genera. Among them, *Proteobacteria* dominated absolutely, with a relative abundance of 66%–99%; followed by *Firmicutes* and *Bacteroidetes*, whose relative abundances increased from 0.11% and 0.29% in the early aging stage to 19.80% and 19.70% in the late aging stage, respectively.

**Fig. 1:**
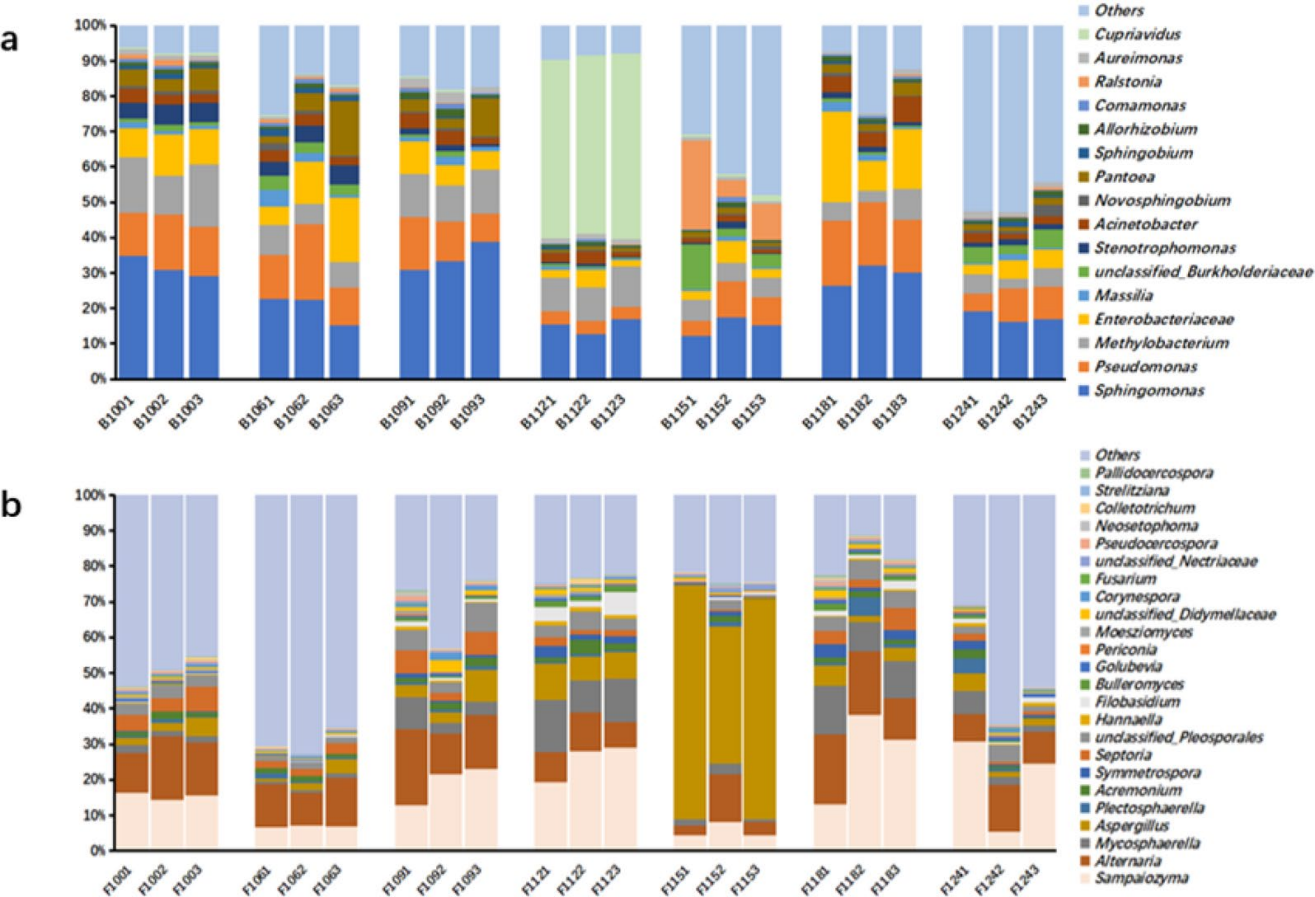
Histogram of bacterial **a** and fimgal **b** composition at genus level of SCTL tobacco during aging. *Note* B and F represent bacteria and fungi respectively. First in the sample number represents SCTL, the second and third numbers represent the length of aging, and the fourth represents the grade of the flue-cured tobacco. 1 represents High-grade flue-cured tobacco, 2 represents medium flue-cured tobacco. 3 represents Low-grade flue cured tobacco

The fungal composition included microorganisms from 18 phyla, 26 classes, 68 orders, 61 families, and 115 genera. The dominant phylum was *Ascomycota*, with a relative abundance of 22.7%–70.4%, followed by *Basidiomycota*, with a relative abundance of 5.9%–47.8%. Figure 1A shows the genus-level composition of dominant bacteria with an average relative abundance > 1%, including *Sphingomonas* (12.1–38.8%), *Pseudomonas* (3.4–21.6%), *Methylobacterium* (2.7–17.5%), *Enterobacteriaceae* (1.8–25.6%), *Pantoea* (0.7–15.3%), and 12 other bacterial genera. "Others" represent the sum of other genera. At the initial stage of aging, the relative abundance of *Sphingomonas* was high (exceeding 30%), and its content was positively proportional to the tobacco grade. However, with the progression of aging, its relative abundance changed with environmental temperature. The relative abundance of *Sphingomonas* peaked at 9 and 18 months of aging, with a significant increase in medium and low-grade tobacco leaves. Similarly, *Pseudomonas* also showed high relative abundances at 0, 9, and 18 months of aging, while the relative abundance of *Methylobacterium* gradually decreased with aging. Additionally, at 12 months of aging, *Cupriavidus* suddenly showed a high abundance (52.5%) in three-grade tobacco leaves; at 15 months of aging, the relative abundance of *Ralstonia* increased, reaching 25.3% in high-grade tobacco leaves, then decreased to a lower level. Fig. 1b shows the composition of dominant fungal genera with an average relative abundance > 1%, including *Sampaiozyma* (4.3–38.2%), *Alternaria* (2.7–21.6%), *Aspergillus* (0.6–65.9%), *Mycosphaerella* (0.7–14.7%), and 8 other genera. "Others" represent the sum of other genera. With the progression of aging, the dominant fungal genera generally showed a fluctuating upward trend, and the relative abundance decreased again at 24 months of aging. *Sampaiozyma* and *Alternaria* had high relative abundances at 0, 9, and 18 months of aging. The relative abundance of *Aspergillus* increased significantly at 15 months of aging, reaching 65.9% in high-grade tobacco leaves.

**Fig. 2:**
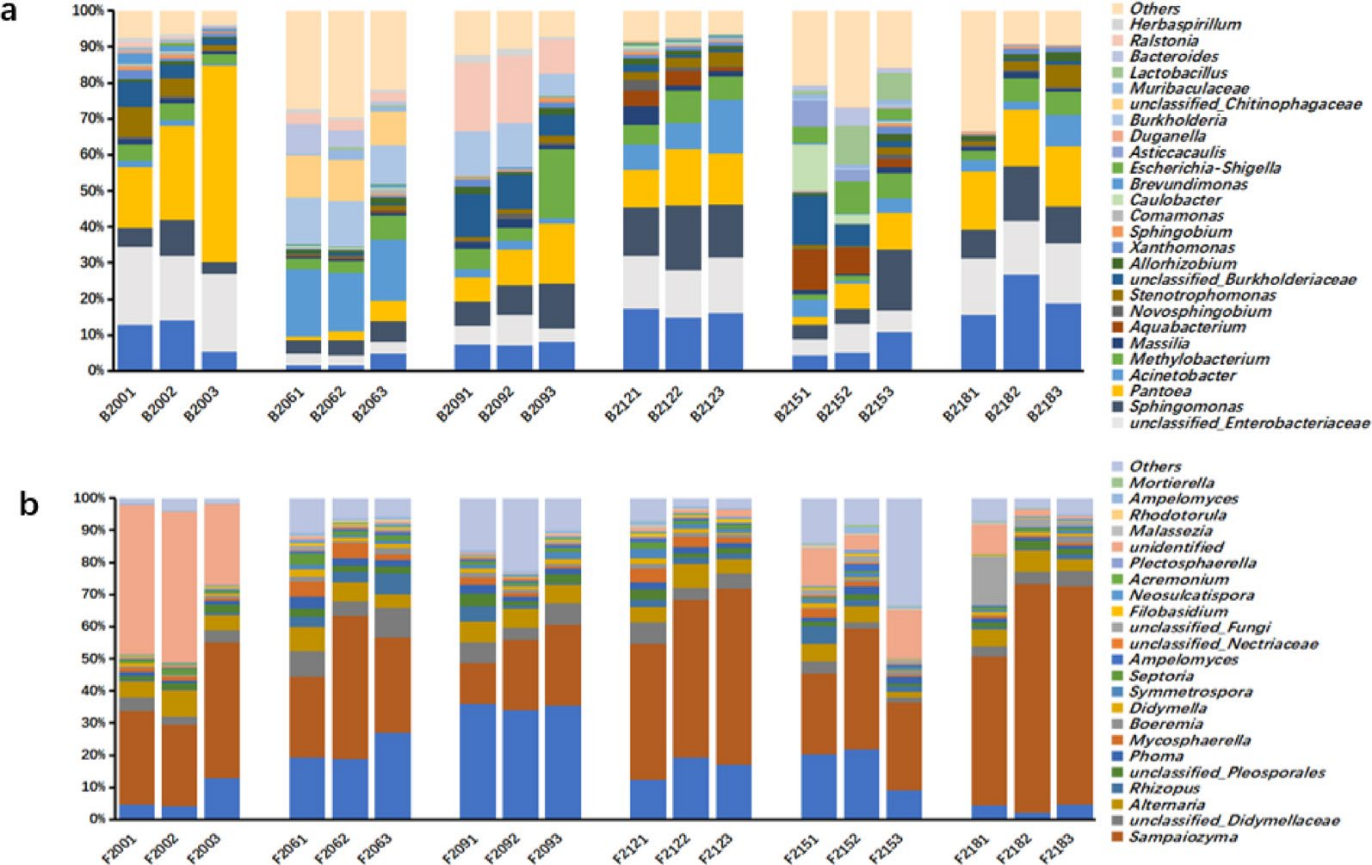
Histogram of bacterial (**a**) and fimgal (**b**) composition at genus level of FCTL during aging. *Note* **B** and **F** represent bacteria and fungi respectively.First in the sample number represents FCTL, the second and third numbers represent the length of aging, and the fourth represents the grade of the flue-cured tobacco. 1 represents High-grade flue-cured tobacco, 2 represents medium flue-cured tobacco. 3 represents Low-grade flue cured tobacco

54 samples of light aromatic tobacco leaves (18 groups, 3 parallel samples per group). High-throughput sequencing yielded 2,829,607 raw bacterial sequences and 3,629,976 raw fungal sequences. After data processing, 2,269,041 valid bacterial sequences and 3,287,042 valid fungal sequences were obtained. According to ASV annotation, *Proteobacteria* dominated the bacterial community with a relative abundance of 59.05% to 98.05%, followed by *Bacteroidetes* (0.20% to 27.23%). In the fungal community, *Ascomycota* accounted for 21.49% to 70.55%, and *Basidiomycota* accounted for 21.73% to 73.19%.The composition of the top 15 bacterial genera by average relative abundance is shown in Fig. 2a, including *Pantoea* (0.9%–54.4%), *Sphingomonas* (3.2%–17.9%), *Methylobacterium* (1.5%–19.2%), *Pseudomonas* (1.7%–26.6%), *Acinetobacter* (0.6%–18.7%), and unclassified *Enterobacteriaceae* (2.5%–21.8%). In the early aging stage, dominant bacterial genera showed higher relative abundances, possibly due to increased microbial activity caused by rising storage temperatures at the start of aging. *Pantoea* had the highest relative abundance (54.4%) in lower-grade tobacco leaves, exhibiting a negative correlation with tobacco leaf grade. As aging progressed and environmental temperature further increased, the relative abundance of *Pantoea* and unclassified *Enterobacteriaceae* continued to rise. At the beginning of aging, *Sphingomonas* and *Pseudomonas* had low relative abundances, which gradually increased over time. By the 18th month, *Pseudomonas* reached a peak of 26.6% in medium-grade tobacco leaves, and *Sphingomonas* peaked at 15.3%.The composition of the top 10 fungal genera by average relative abundance is shown in Fig. 2b. *Sampaiozyma* showed a fluctuating increase in relative abundance, rising from 42.1% in the early aging stage to 71.1% at the end of aging. *Aspergillus* reached its peak relative abundance in the 9th month of aging, while *Alternaria* maintained a low relative abundance throughout the aging process. Overall, except for the lower-grade tobacco leaves (Q153) at the 15th month of aging, the relative abundances of dominant genera in other samples showed a significant negative correlation with tobacco leaf grade.

Studies on microbial diversity in tobacco leaves of different flavor types and varieties have reported varying results. Using the same sequencing technology, Zhang et al*.* (Zhang et al. 2023b, a, c; Ye et al. 2017) investigated the microbial diversity of tobacco leaves stored in different positions of aging warehouses. While the dominant microbial structures at the genus level differed from this study, similar results were observed at the family level and above, with comparable trends in relative abundance changes during aging. At the genus level, most dominant bacteria showed a trend of first decreasing and then increasing in relative abundance, whereas dominant fungi exhibited a trend of first increasing and then decreasing.In contrast, Ye and Wang found that *Bacillus* was a dominant genus in tobacco leaf samples before and after aging, with a relative abundance higher than 20%, while *Sphingomonas*, *Pseudomonas*, and *Pantoea* had lower relative abundances (both below 10% before and after aging)( Ye 2017, Wang et al. 2018). This differs from our findings, where *Sphingomonas*, *Pseudomonas*, and *Pantoea* were dominant genera and *Bacillus* had a relative abundance below 10%. The reasons for these differences mainly lie in two aspects. Ye's research mainly focuses on before and after threshing and redrying, with completely different processing techniques and long-term aging. In Wang's research, the tobacco leaves also adopted the natural aging process, but the tobacco varieties and planting areas he studied are completely different from our samples, which is also the reason for the differences between the research results of these two people and this study. Zhou Jiaxi studied the microbial diversity of tobacco leaves from different aging warehouse sources and found that although the structure of dominant genera had certain deviations from the results of this study, similar results were presented at the family level and above. The trend of abundance changes during the aging process was also similar. The abundance of most dominant bacterial genera first decreased and then increased, while that of dominant fungal genera first increased and then decreased. The common dominant genera present in different tobacco leaf samples can be considered, to a certain extent, as the most core microbial groups that can affect the quality of tobacco leaves during the aging process (Zhou et al. 2018). Collectively, varying research results indicate that production areas, storage locations, and tobacco varieties all influence microbial diversity, yet certain shared dominant genera consistently exist across these tobacco leaf samples.

### Association analysis of dominant species

**Fig. 3:**
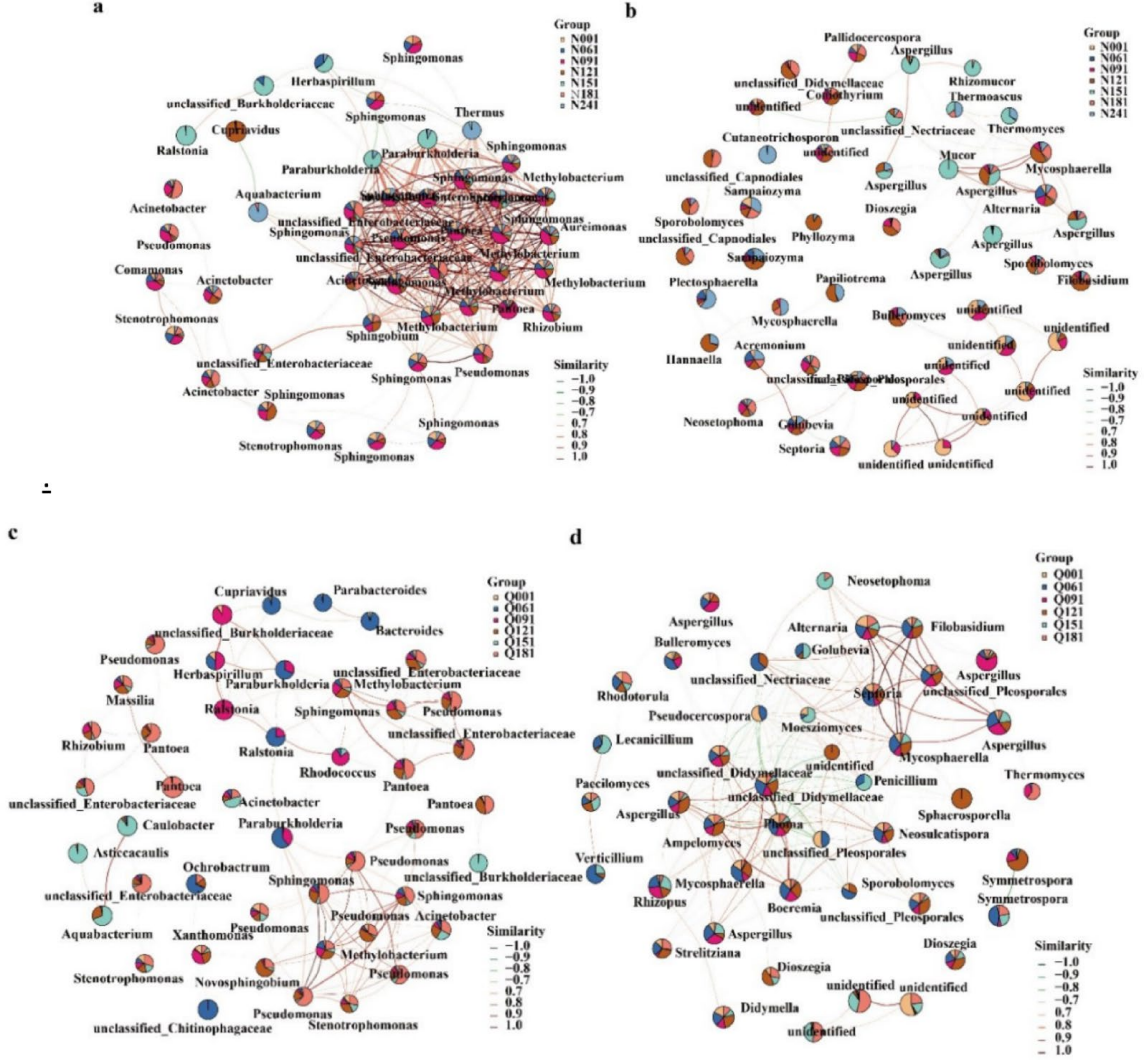
Network diagram of dominant species interactions. *Note* a is bacteria of SCTL; b is fungi of SCTL; c is bacteria of FCTL; d is fungi of FCTL

The close relationship between dominant species indicates a high degree of mutual influence among bacteria, while the relationship with other less closely related species suggests that the fluctuation of this bacterium will not cause too much disturbance to the overall flora(Mao et al. 2024).Through correlation analysis, we explored the co-occurrence or mutual exclusion relationships among the top 50 ASVs (Amplicon Sequence Variants) in terms of relative abundance, as driven by environmental factors during the alcoholization process. Since tobacco leaves of different grades have similar environmental conditions and dominant bacterial genus compositions, this study only presents the correlation relationships between bacterial and fungal communities in high-grade tobacco leaves (Fig. 3). The ASVs (species) in the samples are represented by nodes in the network diagram. The size of the nodes is proportional to the abundance of the ASVs (in log2 (CPM/n) units), and pie charts are used to show the proportion of the relative abundance of these ASVs in different samples. The color of the lines between nodes indicates the correlation between two strains: red lines represent positive correlations, and green lines represent negative correlations. The thickness of the connecting lines indicates the strength of the correlation.

In SCTL the association between dominant bacteria is the closest, among which *Sphingomonas*, *Methylobacterium*, *Pseudomonas* and other genera are closely related. The association between dominant fungal genera is less close than that between bacteria. Among them, *Aspergillus*, *Alternaria*, and *Mucor* are more closely related, while *Aureobasidium pullulans* has a weak association with other genera.In FCTL the closely related genera among dominant bacteria include *Sphingomonas*, *Methylobacterium*, *Pseudomonas*, *Novosphingobium*, etc., while *Pantoea* has a weak relationship with other genera. The dominant fungal flora has a closer relationship than that in SCTL, and *Sampaiozyma* has a loose relationship with other bacteria. At present, there are few studies directly comparing the correlation analysis of microorganisms between SCTL and FCTL, and more research results are concentrated in cigar tobacco leaves. Lin xiaolu studied and analyzed the surface and internal microbial community structures of cigar tobacco leaves at different fermentation stages, and found that the symbiotic network of microorganisms on the surface of cigar tobacco leaves is more complex than that inside, the symbiotic network of bacteria is more complex than that of fungi, and the complexity of the bacterial network is significantly higher than that of fungi (Lin et al. 2025). The research results of Wang, H. et al. indicate that bacterial microorganisms were more closely related to key VFCs and favored a positive correlation, proving that the correlation among bacteria is stronger. At the same time, through the combined analysis of metabolomics and microbiomics, it was found that the correlation between bacterial microorganisms and key flavor substances is more significant (Wang et al. 2024). These research results are consistent with the conclusions drawn in this study.

### Microbial community functional analysis

**Fig. 4:**
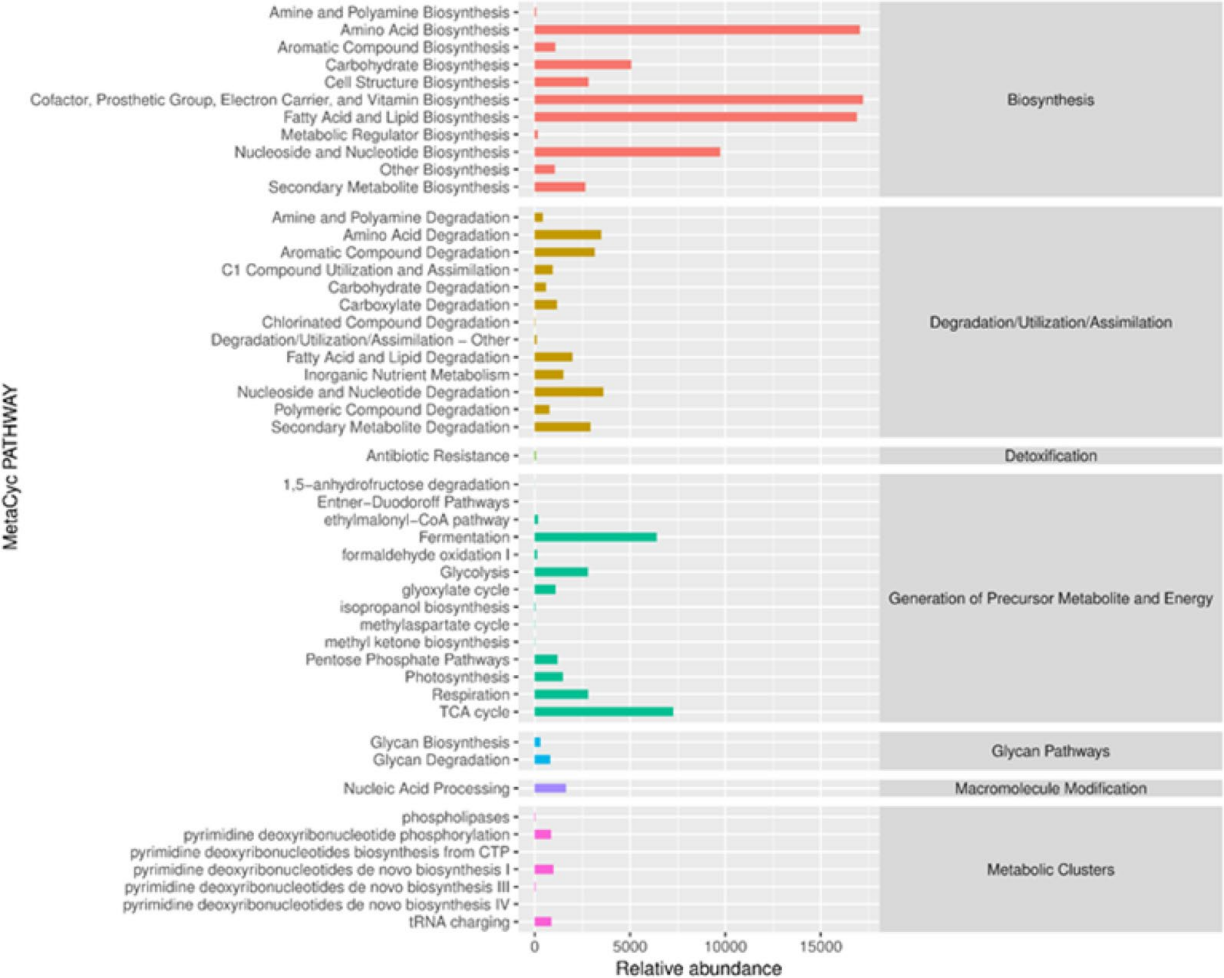
Predicted functional pathway abundance map of MetaCyc secondary metabolic pathways in bacteria

**Fig. 5:**
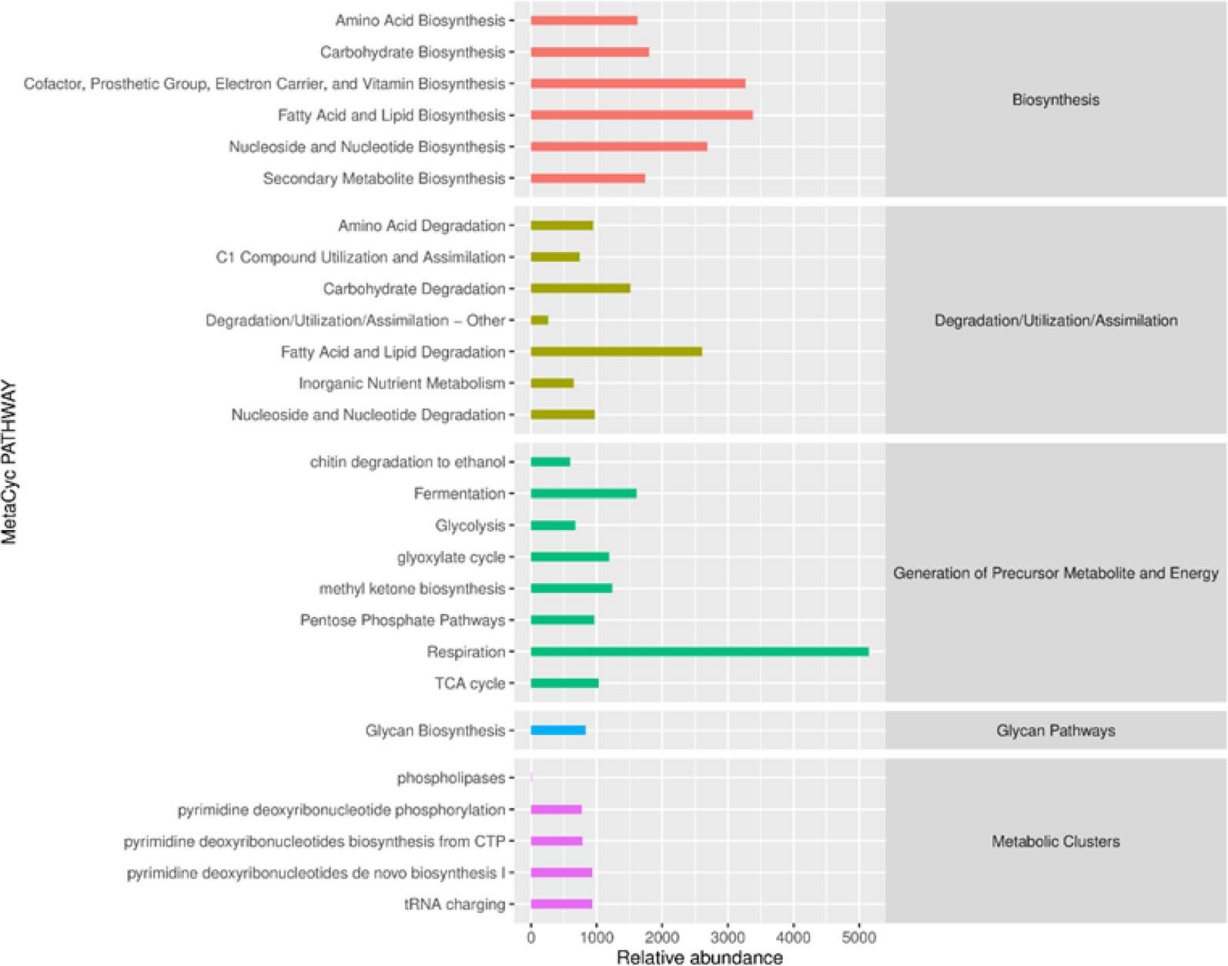
Predicted functional pathway abundance map of MetaCyc secondary metabolic pathways in fungi

PICRUSt2 is a software that predicts the abundance of sample functions (gene families) based on the relative abundances of marker genes in 16S rRNA sequences and ITS sequences. Compared with the first-generation version, PICRUSt2 can use the functional database MetaCyc to predict metabolic pathways. The reference genome data of this database has expanded more than 10 times compared to the original, and the prediction results can be directly compared with the results of metagenomic data ( R Caspi et al. 2012). MetaCyc involves various pathways involved in primary and secondary metabolism, as well as information on their related metabolites, biochemical reactions, enzymes, and genes. Based on the MetaCyc database, PathoLogic in Pathway Tools is used to predict metabolic networks from the annotation of known biological genomes, and classify the metabolic processes of all life in the microbial community. This study predicted that the secondary metabolic pathways of MetaCyc in samples with different flavor types and grades were similar, and their abundance is shown in Fig. 4 and 5. During the aging process, the functional categories and abundance of bacteria consistently exceeded those of fungi, indicating that bacterial communities play a dominant role in the system. Amino acid metabolism is related to microbial growth and also provides necessary precursor substances for the synthesis of secondary metabolites. The degradation products of aromatic amino acids and the further degradation of secondary metabolites contribute to the formation of flavor and aroma substances in tobacco leaves. Shen han's research results show that the proportion of proline in the total amount of free amino acids is extremely significantly positively correlated with the sensory quality (Shen et al. 2022). Tobacco leaf microorganisms show a high abundance in amino acid metabolism. Different microbial strains exhibit a high relative abundance in metabolic pathways related to the synthesis or degradation of different amino acids. For example, *Sphingomonas*, *Pseudomonas*, and *Cupriavidus* show a high functional abundance in the biosynthetic pathway of super amino acids. The important role of bacteria in the metabolism of organic acids during the fermentation process. The research results of Hongyang Si show that among the correlations, *Delftia*, *Ochrobactrum*, *Stenotrophomonas*, and *Rhodococcus* showed negative correlation with oxalic acid, while *Sphingomonas*, *Pseudomonas*, *Methylobacterium*–*Methylorubrum*, and *Staphylococcus* exhibited a positive correlation with oxalic acid. Oleic acid and linoleic acid in tobacco leaves can increase irritation and reduce the smoothness of smoke. (Hu et al. 2023). Lipid substances in tobacco leaves are also an important class of flavor precursor substances. Through oxidation and degradation, they can form aldehydes and ketones with flavors, promoting the improvement of tobacco leaf quality. Hao Jie used GC-IMS technology to identify volatile flavor substances in tobacco. A total of 125 volatile flavor substances were detected, and aldehydes, ketones, alcohols, and esters were the main volatile flavor substances (Hao et al. 2023). Among fungi, *Sampaiozyma*, *Aspergillus*, and some unidentified fungi show a high abundance in fatty acid metabolic functions. Zhang Qing through the study of microbial and enzymatic changes during the air-drying and fermentation processes of cigar tobacco leaves, it was found that Pseudomonas, Monographella, Aspergillus, etc. are key microorganisms. Fungi mainly degrade lignin, cellulose, and pectin through saprophytic action, and at the same time participate in the lipid metabolism process, affecting the formation of tobacco aroma substances (Zhang et al. 2023b, a, c). It can be seen that the growth and metabolic activities of dominant genera directly affect the chemical composition in tobacco leaves and contribute to the formation of flavor substances.

### Dynamic analysis of enzyme activity changes during tobacco leaf aging

**Fig. 6:**
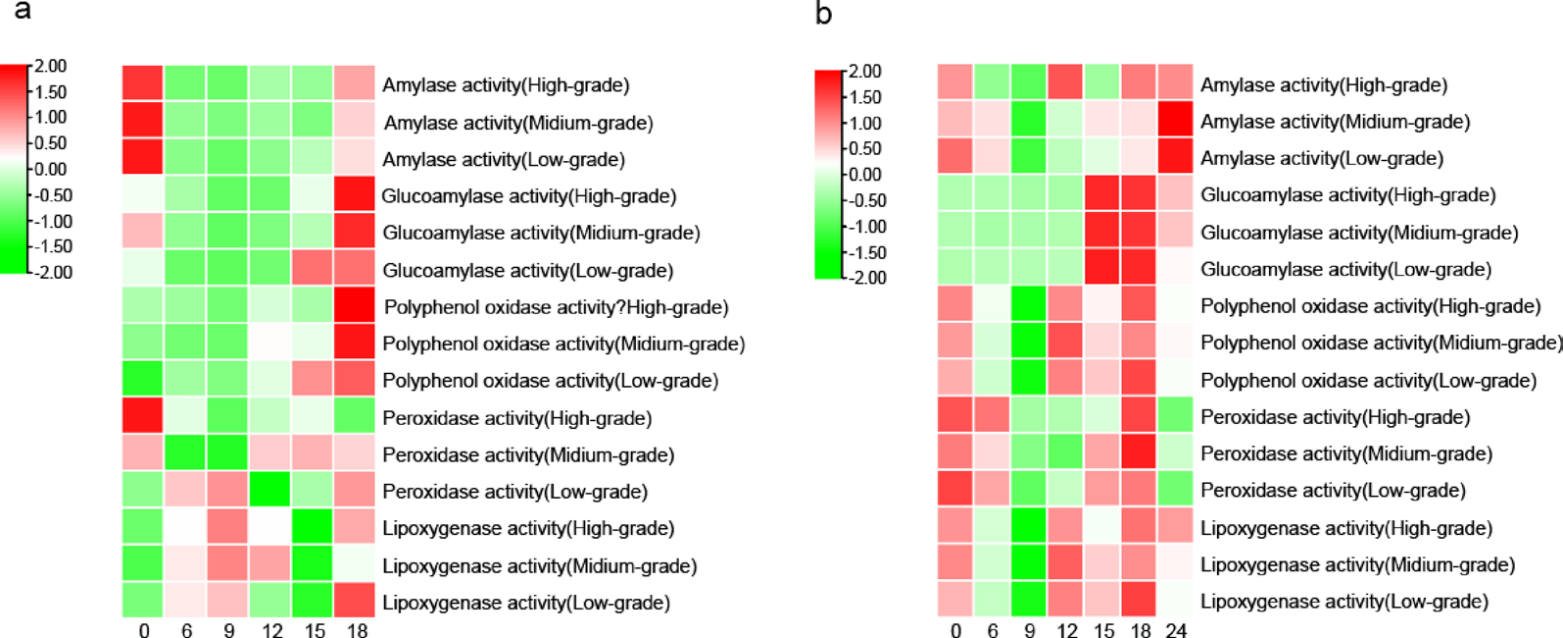
Dynamic analysis of main enzymes activities in SCTLand FCTL during aging process. *Note* **a** SCTL; **b** FCTL

During the aging process, the enzymes in tobacco leaves mainly consist of plant enzymes that retain partial activity after the redrying process and active enzymes secreted by microbial metabolism in tobacco leaves. Different enzymes exhibit variations in functions and substrate specificities during aging. This study investigated amylase and glucoamylase related to starch degradation, as well as lipoxygenase, polyphenol oxidase, and peroxidase related to the degradation of polyphenols and terpenoids in tobacco leaf samples of different flavor types. The changes in enzyme activities during aging are shown in Fig. 6. The differences in the types and activities of the studied enzymes should be related to the variations in tobacco varieties and their microbial diversity (Zhou et al. 2018).

**Fig. 7:**
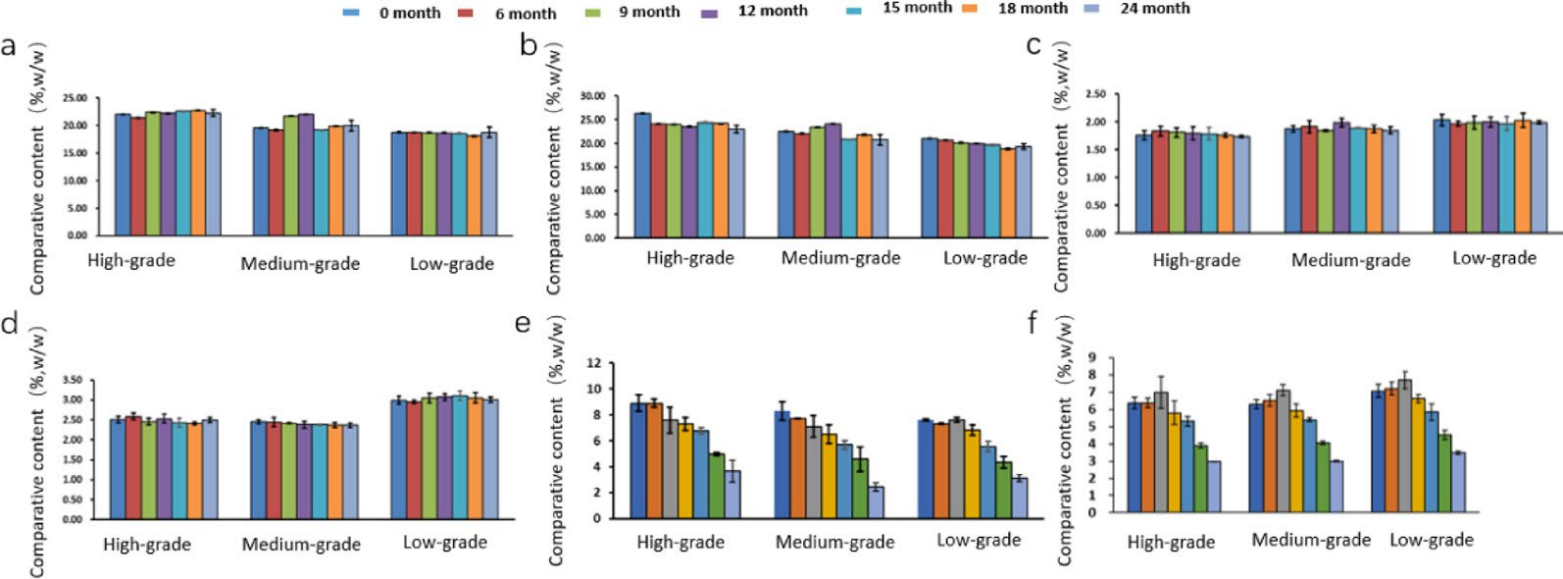
Analysis of routine chemical components in SCTL. *Note* **a** reducing sugar; **b** total sugar; **c** total nitrogen; **d** total plant alkaloids; **e** total phenols; **f** starch

The enzyme activity in tobacco leaves is the comprehensive result of plant enzymes remaining after redrying and enzymes secreted by microbial metabolism. As shown in Figs. 6 and 7, the activities of amylase and glucoamylase in tobacco leaves during aging generally exhibited an overall trend of first decreasing and then increasing. Increased amylase activity correlated with *Bacillus* enrichment, suggesting starch degradation drives flavor precursor formation. The decline in enzyme activity in the early stage of aging is estimated to be related to the gradual weakening of residual activity of endogenous plant enzymes (Wu et al. 2010; Sun et al. 2008). With the enhancement of microbial activity, enzyme activity showed a gradual upward trend in the later stage. Most samples began to increase at 12 or 15 months of aging, and most enzyme activities reached relatively high levels at 18 months of aging. This result is consistent with the findings of Zhou Jiaxi (Zhou et al. 2018) on enzyme activities in tobacco leaves from different aging warehouses. Lipoxygenase is associated with the degradation of fatty acids and carotenoids in tobacco leaves (Yuan et al. 2020; Li et al. 2021), so lipoxygenase plays an important role in aroma enhancement and irritation reduction of tobacco leaves during aging. The lipoxygenase in the two flavor-type tobacco leaves showed a fluctuating trend of first increasing, then decreasing, and then increasing again. From the initial stage of aging to 12 months, the lipoxygenase activity in FCTL of different grades was higher than that in SCTL, after which the enzyme activity in SCTL became higher than that in FCTL. Lipoxygenase in SCTL showed a fluctuating upward trend during aging, reaching a peak at 18 months, followed by a rapid decline at 24 months, with higher activity in medium-grade leaves. In FCTL, lipoxygenase first increased and then decreased during aging, reaching a peak at 18 months, and showed a negative correlation with tobacco grades in the early stage. Polyphenol oxidase and peroxidase are important enzymes in the degradation or transformation of phenolic compounds. The activity of polyphenol oxidase in SCTL was higher than that in FCTL, and it also showed a positive correlation with tobacco grades in the initial stage. The content of peroxidase was comparable in the two types of tobacco leaves, but the content in low-grade FCTL remained at a relatively high level. The fluctuating patterns of various enzymes during aging indirectly reflected the contribution of microbial community metabolism to enzyme activities in tobacco leaves.

### Dynamic analysis of conventional components in tobacco leaves during aging process

**Fig. 8:**
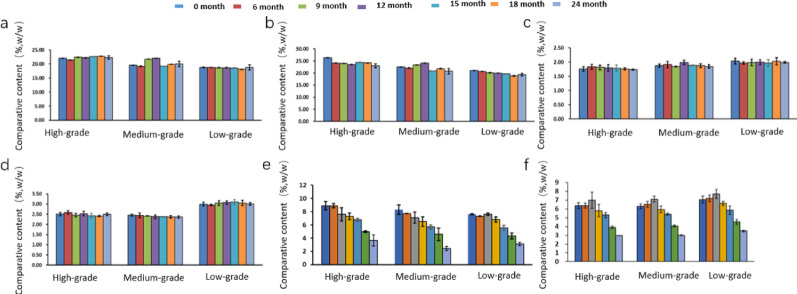
Analysis of routine chemical components in FCTL. *Note* **a** reducing sugar; **b** total sugar; **c** total nitrogen; **d** total plant alkaloids; **e** total phenols; **f** starch

Figures 7 and 8 show the content changes of conventional components in tobacco leaves of different flavor types and grades during the aging process. As indicated by the figures, the contents of total phenols and starch in all tobacco leaf samples decreased significantly, while the changes in other components were not obvious. Phenolic compounds are important aroma precursor substances in tobacco leaves, among which chlorogenic acid and rutin account for 80% of the total. The decrease in total phenol content is accompanied by an increase in related aroma components. The total phenol content in FCTL was higher than that in SCTL at the early stage of aging, and the two became comparable in the later stage. The contents of both flavor types of tobacco leaves showed a slight increase in the early stage of aging, followed by a gradual decrease. This is because most phenolic substances in tobacco leaves exist in the conjugated form with glycosides or esters. During the aging process, under the action of hydrolases, they are converted into free polyphenols that can be further degraded, leading to a slight increase in content, which then decreases due to the degradation of phenolic substances. This is consistent with the research results of Liang. The relative content of polyphenols in tobacco leaves decreased significantly during the aging period of 9–27 months, and then changed slightly. (Liang 2023). The starch content continuously decreased during the aging process, and the content was generally positively related to the tobacco grade. The starch content in SCTL was higher than that in FCTL. At the end of aging, both decreased to about 3%, which meets the requirement for starch content in tobacco raw materials for cigarette production. The degradation of starch forms reducing sugars, which promotes the enhancement of sweetness and aftertaste of tobacco leaves, and continuously improves the quality of tobacco leaves. The contents of reducing sugars and total sugars in both flavor types of tobacco leaves were positively related to the grade, but the contents of reducing sugars and total sugars in FCTL were significantly higher than those in SCTL, indicating that the increase in sugar content helps to increase the mellow sweetness of tobacco leaves and endows light-aroma tobacco leaves with more obvious characteristics (Liu et al. 2020). However, the contents of total sugars and reducing sugars showed fluctuating patterns during the aging process, which is consistent with the research results of Gong Xiaowei, Liu, Hongguan and Hu Yajie. (Gong et al. 2010a, b; Liu et al. 2015a, b; Hu et al. 2020), but inconsistent with the results of Zhang Qingming and Fan Jianqiang. (Fan et al. 2004; Zhang et al. 2014), which may be related to the degradation of total sugars and the consumption of reducing sugars by microorganisms. The contents of total nitrogen and total plant alkaloids in the two flavor types of tobacco leaves were comparable, and the changes during the aging process were not significant. The total nitrogen and total plant alkaloids in SCTL showed a slight decrease, while those in FCTL showed a slight increase, which is consistent with the research results of Zhang Qingming. on tobacco leaves of different varieties. The total nitrogen content showed an inverse relationship with the tobacco grade, and the content of total plant alkaloids was the highest in low-grade tobacco leaves, indicating to a certain extent that the degradation of total nitrogen and plant alkaloids is conducive to the improvement of tobacco leaf quality, which can reduce the irritation and harshness of tobacco leaves (Li et al. 2022).

### Correlation analysis of key factors in the aging process

To explore the mechanism of the aging process, this study constructed the associative relationships among various factors with the aid of RDA (Redundancy Analysis). During the aging process, microbial metabolism and enzymatic actions degrade or transform substance components, forming key substance components that exert important influences on quality, thereby affecting the quality of tobacco leaves. The redundancy analysis effects among key factors of SCTL and FCTL are shown in Table 3.

**Table 3:** Redundancy effect of correlation between dominant microorganism and main enzyme activities in flue-cured tobacco during aging process

Sample	Variable	Contribution rate/%	F value	*P* value	Adjustment *P* value
SCTL	Bacteria and enzyme activity		16.5	0.002	
	Bacteria and lipoxygenase	23.6	10.7	0.004	0.02
	Bacteria and amylase activity	20.3	8.8	0.004	0.02
	Bacteria and peroxidase activity	15.3	4.8	0.004	0.02
	Bacteria and glucoamylase activity	13.5	4.2	0.004	0.02
	Fungi and enzyme activity		14.2	0.002	
	Fungi and glucoamylase	39.4	6.7	0.002	0.01
	Fungi and lipoxygenase	15.6	2.7	0.04	0.02
	Bacteria and conventional components		3.6	0.02	
	Bacteria and total phenols	18.6	4.9	0.012	0.04
	Fungi and conventional components		3.6	0.002	
	Fungi and reducing sugars	10.4	2.5	0.008	0.024
FCTL	Bacteria and enzyme activity		29.6	0.002	
	Bacteria and amylase activity	57.0	17.7	0.002	0.01
	Bacteria and glucoamylase activity	33.4	10.1	0.002	0.01
	Bacteria and polyphenol oxidase activity	26.9	9.7	0.002	0.01
	Fungi and amylase activity		23.6	0.002	
	Fungi and lipoxygenase	53.7	15.2	0.002	0.01
	Fungi and amylase activity	32.8	9.5	0.002	0.01
	Bacteria and conventional components		4.1	0.002	
	Bacteria and total phenols	28.1	4.1	0.008	0.048
	Fungi and conventional components		2.5	0.002	
	Fungi and starch	53.5	6.9	0.008	0.048

**Fig. 9:**
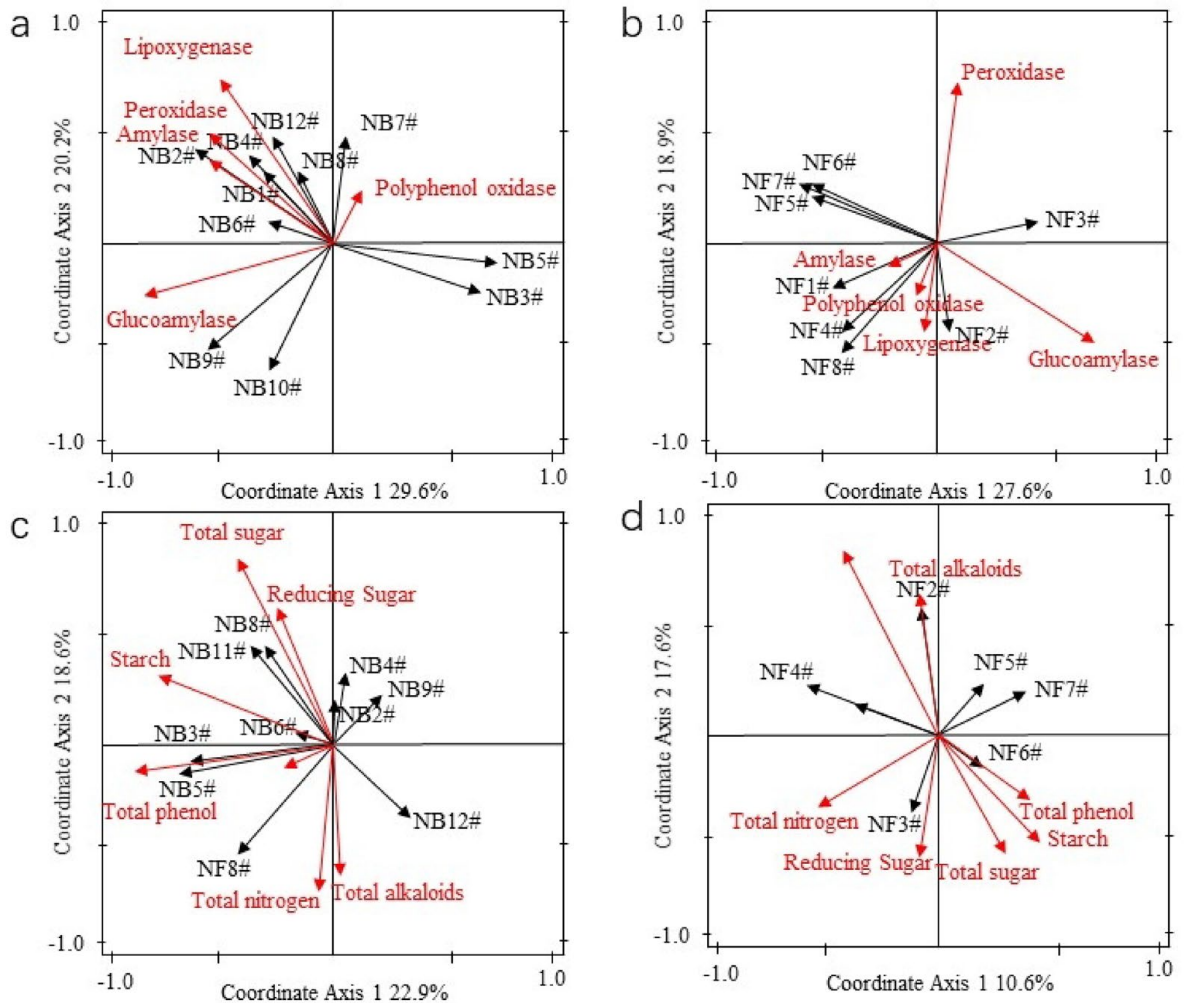
Redundancy analysis between dominant microbial genera and enzyme activities of SCTL during aging process. *Note* **a** dominant bacterial genus—enzymatic activity; **b** dominant fungal genus—enzymatic activity; **c** dominant bacterial genus—conventional components; **d** dominant fungal genus—conventional components

**Fig. 10:**
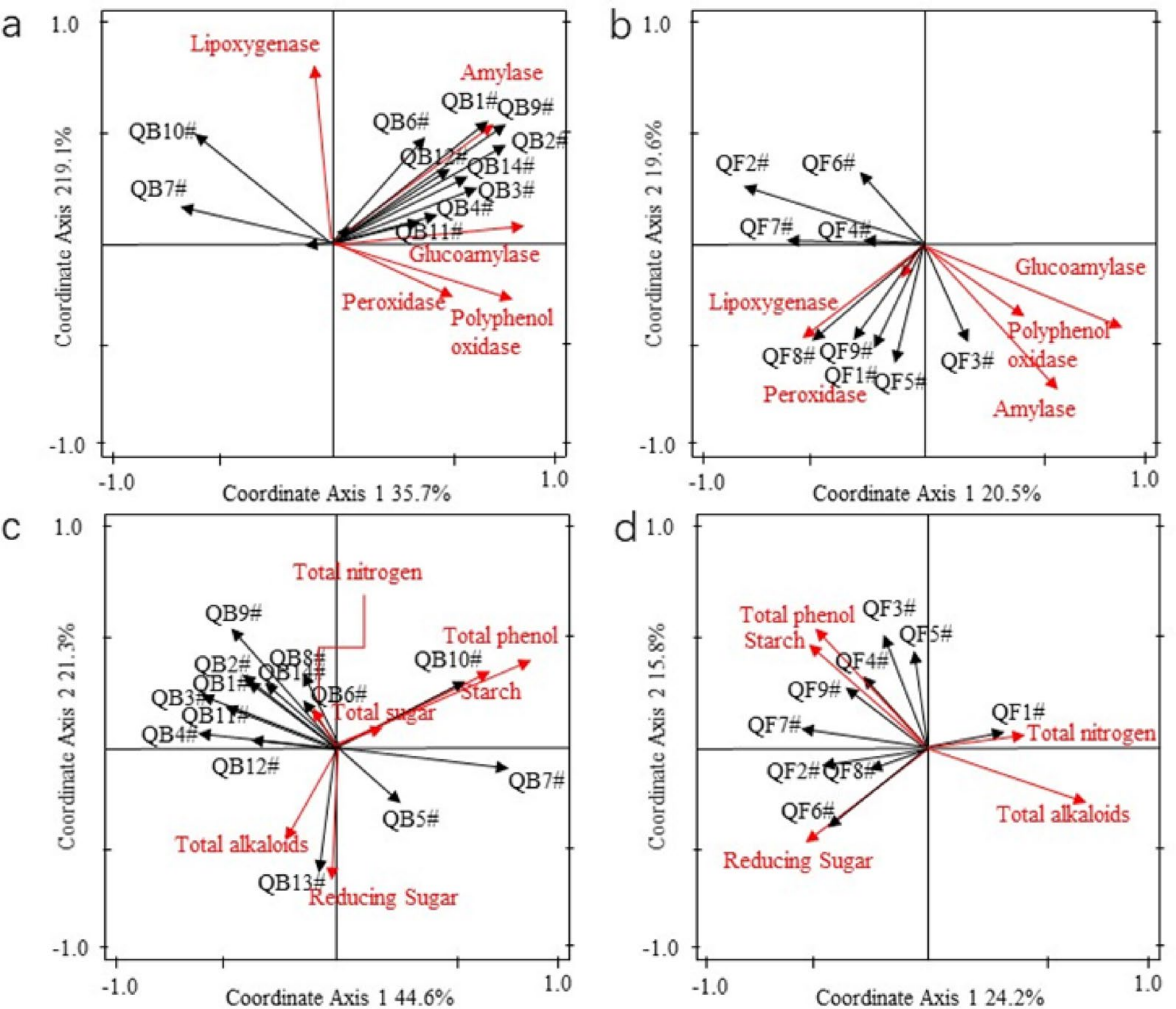
Redundancy analysis between dominant microbial genera and enzyme activities of FCTL during aging process. *Note* **a** dominant bacterial genus—enzymatic activity; **b** dominant fungal genus—enzymatic activity; **c** dominant bacterial genus—conventional components; **d** dominant fungal genus—conventional components

In two types of tobacco leaves, there is a significant positive correlation between enzymes and most dominant bacterial genera (Figs. 9 and 10). Among these enzymes, lipoxygenase (LOX) contributes the most, followed by amylase, and both have the closest relationship with changes in the relative abundance of bacterial genera. Specifically, *Pseudomonas* (NB2), Bacillus (NB12), *Acinetobacter* (NB7), unclassified *Burkholderiaceae* (NB9), *Stenotrophomonas* (QB9), *Pantoea* (QB1), unclassified *Enterobacteriaceae* (QB2), and *Bacillus* (QB14) show positive correlations with enzymes such as lipoxygenase, amylase, peroxidase, and glucoamylase. Studies have shown that in Indonesian cigar tobacco leaves, the genus *Pseudomonas* has a relatively high abundance, and its metabolites may enhance LOX activity through a similar mechanism, thereby promoting the degradation of carotenoids to produce aroma substances such as solanesone. Metabolites of *Bacillus* (Nα-acetyl-L-lysine) may activate host LOX homologous proteins (Loxl2) to generate H₂O₂, which in turn regulates the redox balance and signal pathways of cell proliferation, thus affecting the functional expression of LOX(Xiao et al. 2024).*Bacillus* is the main source of amylase in tobacco leaves, which significantly improves the smoothness and aftertaste of cigarette smoke. It accelerates the conversion of starch into carbohydrates by secreting amylase, providing precursors for the synthesis of aroma substances. *Pseudomonas* can secrete amylase, protease, and cellulase simultaneously, which can reduce starch content and increase the content of aroma substances such as ketones and esters (Shang 2025).The abundance of *Acinetobacter* increases in the late stage of tobacco leaf aging, and its metabolic activities are closely related to the production of carbonyl compounds (aldehydes and ketones). The precursors of carbonyl compounds may be derived from starch degradation products; therefore, *Acinetobacte*r may indirectly affect the function of amylase by regulating the metabolic network (Jin 2022).

In SCTL, the activities of glucoamylase and lipoxygenase (LOX) show a significant correlation with the changes in the abundance of dominant fungal genera, and exhibit a relatively close positive correlation with *Aspergillus* (NF3), *Alternaria* (NF2), *Filobasidium* (NF8), *Sampaiozyma* (NF1), and *Mycosphaerella* (NF4).*Aspergillus* can secrete enzymes such as α-amylase, glucoamylase, and cellulase to targetedly decompose macromolecular carbohydrates (starch and cellulose) in tobacco leaves (Gong 2025). Additionally, the acid proteases and neutral proteases secreted by *Aspergillus* can break down crude proteins in tobacco leaves into free amino acids. These free amino acids not only serve as precursors for aroma substances but also reduce the "ammonia odor" and irritation caused by protein combustion. Furthermore, *Aspergillus* participates in the transformation of phenols and lipids in tobacco leaves through polyphenol oxidase, lipase, and lipoxygenase (LOX), thereby affecting tobacco leaf color (the formation of brown color) and the regulation of astringency (Dong 2025). *Sampaiozyma* secretes a variety of enzymes to targetedly decompose macromolecular substances in tobacco leaves while producing key aroma precursors, which contributes to the maturation of tobacco leaves. It may secrete nicotine dehydrogenase to convert nicotine into nornicotine, thereby reducing the irritation of cigarette smoke (Liu et al. 2024; He et al. 2024). Additionally, *Sampaiozyma* can secrete carotenoid cleavage dioxygenase (CCD) to convert β-carotene, lutein, and other carotenoids into key aroma substances such as β-damascenone and ionone (Huang et al. 2022).

In FCTL, lipoxygenase (LOX) and amylase show a significant correlation with changes in the abundance of dominant fungal genera. Among these fungi, *Septoria* (QF8) and *Filobasidium* (QF9) have a close relationship with lipoxygenase.*Filobasidium* exhibit significant LOX activity, which can catalyze the oxidative degradation of unsaturated fatty acids (linolenic acid and linoleic acid) in tobacco leaves, generating green leaf volatiles (GLVs) such as hexanal and leaf alcohol—these substances contribute to the honey-sweet and green grass-like flavors of tobacco leaves (Wu et al. 2023). The peroxides produced by LOX catalysis can activate terpene synthases (geranylgeranyl pyrophosphate synthase), promoting the production of terpene-derived aroma substances like geranylacetone and β-damascenone. Although *Filobasidium* does not directly secrete amylase, its metabolites can regulate the environmental pH, thereby enhancing the amylase activity of bacteria such as *Bacillus* (Jia et al. 2023).

As shown in Fig. 10c–d, total phenols and starch made greater contributions to the abundance changes of dominant bacterial and fungal genera, respectively. Among them, *Ralstonia* (QB10) and *Burkholderia* (QB7) were closely related to total phenol content. *Ralstonia* is widely present in plants and can stimulate plants to enhance the activity of polyphenol oxidase to resist viral attacks, thereby affecting polyphenol content (Zou et al. 2022). At present, our research only involves the detection of conventional chemical components. In subsequent studies, we can utilize three non-targeted detection technologies based on the UPLC-QTOP-MS, GC-TOP-MS derivatization, and HP-SPME-GC-TOP-MS detection platforms to conduct multi-dimensional analysis of non-volatile and volatile components in the samples. This will enable a comprehensive and systematic analysis of the differential compounds and their differential metabolic pathways before and after alcoholization, further identifying the key factors affecting the quality changes of tobacco leaves during the alcoholization process.

## Conclusions

This study focused on the aging process of SCTL and FCTL, systematically analyzed the dynamic changes of microbial diversity, enzyme activity, and conventional components, and revealed the interaction mechanism among microorganisms, enzymes, and conventional chemical components.

Analysis of the diversity in tobacco leaves of different flavors and grades revealed the common dominant bacterial genera with a relative abundance greater than 1%. Among them, the bacteria include *Sphingomonas*, *Pseudomonas*, *Methylobacterium*, *Acinetobacter*, and the fungi include *Alternaria*, *Aspergillus*, *Sampaiozyma*. Through the predictive function of the microbial community, it is known that among bacteria, pathways related to the formation of tobacco leaf flavor substances, such as amino acid synthesis and degradation, aromatic amino acid degradation, fatty acid synthesis, secondary metabolite degradation, carbohydrate degradation and fermentation, etc., and among fungi, pathways such as fatty acid degradation, all have relatively high abundances.

A correlation analysis was conducted on the key factors affecting quality in the two aroma types. Using the cumulative explanatory rate of the RDA ordination diagram and the contribution analysis of factors with significant impacts, the combined effect of all factors' total variables in the two aroma types of tobacco leaves was significant. Among them, in the SCTL, the significant factors with the highest contribution rates to the changes in the abundance of dominant bacteria and fungi were lipoxygenase and glucoamylase, respectively; in the FCTL, the significant factors with the highest contribution rates to the changes in the abundance of dominant bacterial and fungal genera were amylase and lipoxygenase, respectively. Through the degradation of starch and polyphenolic substances, they promote the degradation of long-chain fatty acids and terpenoids, as well as the formation of Maillard reaction products.

These insights support targeted microbial inoculation and precision-controlled aging processes. Based on the research of tobacco leaf aging mechanism, high—throughput screening technology is used to obtain strains with specific functions from the tobacco leaf mixed—bacteria system. Based on the enzyme—producing ability, strains with the functions of degrading macromolecular substances and promoting aroma production are obtained. According to the corresponding enzyme—producing ability of the strains, the quality—improving ability of tobacco leaves and the growth performance of the strains themselves, the functional strains that can be used subsequently are determined. During the industrial production of tobacco leaf aging, by inoculating these functional microorganisms into tobacco leaves and precisely controlling factors such as the inoculation amount of these strains and the growth environment, the directional accumulation of characteristic aroma substances in tobacco leaves is promoted, and the precise regulation of tobacco leaf quality is achieved.

## Data Availability

When it is necessary for the article to be published, it can be provided.
